# Cross sectional study of multiresistant bacteria in Danish emergency departments: prevalence, patterns and risk factors for colonization (AB-RED project)

**DOI:** 10.1186/s12873-018-0178-1

**Published:** 2018-08-20

**Authors:** Christian B. Mogensen, Helene Skjøt-Arkil, Annmarie T. Lassen, Isik S. Johansen, Ming Chen, Poul Petersen, Karen V. Andersen, Svend Ellermann-Eriksen, Jørn M. Møller, Marc Ludwig, David Fuglsang-Damgaard, Finn Nielsen, Dan B. Petersen, Ulrich S. Jensen, Flemming S. Rosenvinge

**Affiliations:** 1 Emergency Department, Hospital of Southern Jutland, Aabenraa, Denmark; 2 Emergency Department, Odense University Hospital, Odense,, Denmark; 3 Department of infectious diseases, Odense University Hospital, Odense,, Denmark; 4 Department of Clinical Microbiology, Hospital of Southern Jutland, Soenderborg, Denmark; 5 Emergency Department, Regional Hospital West Jutland, Herning, Denmark; 6 Emergency Department, Aarhus University Hospital, Aarhus, Denmark; 7 Department of Clinical Microbiology, Aarhus University Hospital, Aarhus, Denmark; 8 Emergency Department, Aalborg University Hospital, Aalborg, Denmark; 9 Emergency Department, North Denmark Regional Hospital, Hjoerring, Denmark; 10 Department of Clinical Microbiology, Aalborg University Hospital, Aalborg, Denmark; 11 Emergency Department, Slagelse Hospital, Slagelse, Denmark; 12 Emergency Department, Zealand University Hospital, Koege, Denmark; 13 Department of Clinical Microbiology, Slagelse Hospital, Slagelse, Denmark; 14 Department of Clinical Microbiology, Odense University Hospital, Odense,, Denmark

**Keywords:** Emergency department, Colonization, Multiresistant bacteria

## Abstract

**Background:**

Multiresistant bacteria (MRB) is an increasing problem. Early identification of patients with MRB is mandatory to avoid transmission and to target the antibiotic treatment. The emergency department (ED) is a key player in the early identification of patients who are colonized with MRB.

There is currently sparse knowledge of both prevalence and risk factors for colonization with MRSA, ESBL, VRE, CPE and CD in acutely admitted patients in Western European countries including Denmark. To develop evidence-based screening tools for identifying carriers of resistant bacteria among acutely admitted patients, systematic collection of information on risk factors and exposures is required. Since a geographical variation is suspected, it is desirable to include emergency departments across the country.

The aim of this project is to provide a comprehensive overview of prevalence and risk factors for MRSA, ESBL, VRE, CPE and CD colonization in patients admitted to Danish ED’s. The objectives are to describe the prevalence and demography of resistance, co-infections, to identify risk factors for carrier state and to develop and validate a screening tool for identification of carriers.

**Methods:**

Multicenter descriptive and analytic cross-sectional survey from January–May 2018 of around 10.000 acutely admitted patients > 18 years in 8 EDs for carrier state and risk factors for antibiotic resistant bacteria. Information about the background and possible risk factors for carrier status together with swabs from the nose, throat and rectum is collected and analyzed for MRSA, ESBL, VRE, CPE and CD. The prevalence of the resistant bacteria are calculated at hospital level, regional level and national level and described with relation to residency, sex, age and risk factors. A screening model for identification of carrier stage of resistant bacteria is developed and validated.

**Discussion:**

The study will provide the prevalence of colonized patients with resistant bacteria on arrival to the ED and variation in demographic patterns, and will develop a clinical tool to identify certain risk groups. This will enable the clinician to target antibiotic treatments and to reduce the in-hospital spreading of resistant bacteria. This knowledge is important for implementing and evaluating antimicrobial stewardships, screening and infection control strategies.

**Trial registration:**

Clinicaltrials.gov: NCT03352167 (registration date: 20. November 2017).

## Background

Multiresistant bacteria (MRB) is an increasing problem in many parts of the world [1]. In Denmark, attention has been focused in particular to methicillin resistant *Staphylococcus aureus* (MRSA), but there has also been an increase in the prevalence of extended-spectrum beta-lactamase-producing enterobacteria (ESBL), vancomycin resistant enterococci (VRE) and carbapenem resistant enterobacteria (CPE) [2].

The consequences of the widespread antibiotic use and thus increasing bacterial resistance to antibiotics are multiple [3]. Both antibiotic-associated diarrhea caused by infections with toxin-producing *Clostridium difficile* (CD) [4] and infections and spread of MRB lead to increased healthcare costs, increased morbidity and mortality [5], and complicate advanced treatments that depend on effective infection control, like treatment of malignancies and transplantations [6, 7].

Early identification of patients colonized with resistant bacteria is mandatory to avoid in-hospital transmission and to target the antibiotic treatment to the individual patient. Antibiotics are used in many areas of the hospitals, but especially the treatment of acutely admitted patients often includes an antibiotic prescription. The emergency departments (ED) are key players in the in-hospital use of antibiotics and in the early identification of patients who are colonized with MRB since the majority of patients are admitted through these departments.

There is currently very sparse knowledge of both prevalence and risk factors for colonization with MRSA, ESBL, VRE, CPE and CD in acutely admitted, unselected patients in Western Europe countries including Denmark. A Danish study has shown that the prevalence of MRSA in an area with ​​presumed high numbers of carriers was 0.9% in the emergency department [8]. The prevalence of the other MRB has yet to be described at the ED level.

The Danish National Board of Health has published guidelines for targeted screening for isolation of MRSA, based on questionnaires concerning exposure factors [9]. This screening is expected to identify only some of the carriers [8]. In order to clarify the extent of the problem and prioritize the preventive response to the spread of MRB, it is crucial to know the prevalence of these bacteria in unselected acutely admitted patients to the Danish EDs. To develop evidence-based screening tools for identifying carriers of resistant bacteria among the acutely admitted patients, systematic collection of information on risk factors and exposures is required. Since there is a certain geographical variation in prevalence of carriers, it is desirable to include emergency departments across the country.

### Aims and objectives

The aim of this project is to provide a comprehensive overview of prevalence and risk factors for MRSA, ESBL, VRE, CPE and CD colonization in patients admitted to Danish emergency departments at a national level.

The objectives are:

I) To describe the prevalence of MRSA, ESBL, VRE, CPE and CD colonization in unselected, acutely admitted patients and the regional, urban/country and other demographic variations.
II) To describe the occurrence of simultaneous patient colonization with two or more resistant bacteria (MRSA, ESBL, VRE, CPE and CD).
III) To identify risk factors for colonization with MRSA, ESBL, VRE, CPE and CD
IV) Based on the identified risk factors, to develop screening tools for identification of carriers.

## Methods

Participants, interventions, and outcomes.

### Trial design

Multicenter descriptive and analytic cross sectional survey of acutely admitted patients for risk factors and carriage of MRB.

### Study setting

The project takes place in emergency departments and departments of clinical microbiology at the university hospitals in Odense, Aarhus, Aalborg, and Køge, and the regional hospitals in Aabenraa, Herning, Hjørring and Slagelse and covers 4 of the 5 Danish regions, and approximately 70% of the Danish population.

### Eligibility criteria

#### The inclusion criteria are

- Patients aged 18 years or above.
- Patients present in one of the participating emergency departments at least 4 h.
- Patients must be mentally competent and able to consent to participate.

#### The exclusion criteria are

- Patients who have been admitted for more than 16 h before enrollment.
- Patients where swabbing in the rectum, nose or throat is not possible due to anatomic or surgical reasons.

### Interventions

There are no interventions in this study.

### Outcomes

The primary outcome is the presence of MRB (MRSA, ESBL, VRE, CPE) and CD in patients in the ED, expressed as prevalence (proportion) in percentage on admission among the included patients.

The secondary outcomes are the information on admission concerning risk factors for carriage of antibiotic resistant bacteria, expressed as the proportion and odds ratios between patients with and without MRB (Table 1).

**Table 1:** Variables examined in the study

Examined risk factors for carrying resistant bacteria	-Travel activity last year -Work/stay at hospital, nursing home, schools, prison, military, asylum center/refugee camp, hostel, daycare or war zone in and outside the Nordic region. -Signs of infections, shared sports equipment, tattoos/piercings -Contact with mink farm, live pigs, cattle or poultry -Hospitalized for the past six months -Invasive surgery performed at a foreign clinic/hospital -Drainage tubes, indwelling catheters, nutrition tubes, ulcers, abscesses, eczema, chronic skin disorders or IV drug abuse within the last six months -Antibiotic treatment within the last 6 months -Previously carrier of resistant bacteria, household-like contact with carrier or worked/stayed in places with known transmission of resistant bacteria
Outcome variables	-MRSA: methicillin resistant *Staphyloccus aureus* -ESBL: extended-spectrum beta-lactamase producing enterobacteria -VRE: vancomycin resistant enterococci (VRE) -CPE: carbapenem resistant enterobacteria -CD: *Clostridium difficile*

### Participant timeline

Enrolment to the study commences from January 2018. Due to local conditions some of the participating departments will include patients from February 2018. Enrollment continues until the predefined sample size for each department has been reached, which is estimated to be no longer than 4 months after the inclusion start in each department. The last patient is expected to be enrolled in the end of June 2018.

### Sample size

A Danish study showed an MRSA occurrence of 0.9% in an ED [8]. It is assumed that VRE and CPE have a lower occurrence and ESBL and CD a higher occurrence [2]. With the funding obtained, 1.325 patients can be included per ED, corresponding to 2650 patient in each region and 10.600 patients in total. The true frequencies of the prevalence of resistant bacteria with 95% confidence, based on expected prevalence of resistant bacteria of 0.5%, 1%, 10% and 25% is shown in Table 2 for different samples sizes (anticipated admission rate of 10.000 patients/year).

**Table 2:** Sample size calculation for prevalence (source Openepi.com)

Calculations of sample size estimates 95% limits of confidence								
	sample size (number of patients)							
	600	1100	1500	1750	2200	3500	5000	10,000
Anticipated prevalence of resistant bacteria	95% limits of confidence (+/− % of prevalence)							
0.5%	+/− 0.5	+/− 0.4	+/− 0.35	+/− 0.30	+/− 0.25	+/− 0.20	+/− 0.14	+/− 0.03
1%	+/− 0.8	+/− 0.55	+/− 0.45	+/− 0.40	+/− 0.35	+/− 0.30	+/− 0.20	+/− 0.05
10%	+/− 2.5	+/− 1.75	+/− 1.4	+/− 1.25	+/− 1.2	+/− 1.0	+/− 0.6	+/− 0.2
25%	+/− 3.5	+/− 2.5	+/− 2.0	+/− 1.75	+/− 1.70	+/− 1.45	+/− 0.9	+/− 0.2
	Example: at a prevalence of for instance MRSA of 0.5% the 95% limits of confidence with a sample size of 1.100 patient is +/0.4%, which means that the prevalence with 95% certainty is from 0.1–0.9%.							

Assumptions: 10.000 patients pr year, 95% confidence limits

For the analysis of risk factors for carrier stage of resistant bacteria, only calculations at national level will be performed.

### Recruitment

All patients in the ED should be contacted as early as possible during the enrollment process by a project employee who informs both verbally and in writing about the project. If the 4 hour requirement is an obstacle for receiving the expected number of inclusions, the steering committee might waive this criterion. The patient is allowed to have a family member, friend or acquaintance, as a lay representative present at the information.

The project employee will have a healthcare background (nurse or medical student) and is employed by the department.

Privacy will be secured during the information and no treatment must be delayed because of the enrolment.

All patients are offered up to 1 hour’s consideration time before written consent is obtained. The consent includes that the project employee is allowed to obtain a swab from pharynx, nose and rectum, that the microbiological analysis and the exposure information is used for a scientific purpose, and that information concerning antibiotic consumption from the patient records is registered. The patient also receives information about who he/she can contact afterwards for additional questions.

### Data collection methods

#### Interview

The project staff in the emergency department collects information about the background and possible risk factors for carrier status through an interview with the patient. The questions included are prepared on the basis of the Danish National Board of Health’s guidance on preventing spreading of MRSA [9].

#### Collection of swabs

The patient is swabbed in the nose (ESwab (Copan, SSI Diagnostica, Hillerød, Denmark)), throat (ESwab) and rectum (FecalSwab (Copan)).

Nasal swabs are obtained by inserting a swab into the anterior nares and rotating it along the mucous membrane. Throat swabs are collected by rubbing a swab along the mucosal surfaces of the tonsils and pharynx. These samples are already collected routinely in Denmark in selected patients who are screened for MRSA, according to the guidelines of the National board of Health.

Rectal swabs are obtained by inserting a swab 1–2 cm. into the anal canal and rotate it against the mucosal surface a few times. The procedure is comparable to a temperature measurement in the rectum.

The three swabs are labeled with barcodes containing information on project department and a unique project running number. All samples are sent to the regional department of clinical microbiology using the same transport arrangements as for routine tests.

#### Microbiological analysis

The collected samples are examined at the Departments of Clinical Microbiology at Aalborg University Hospital, Aarhus University Hospital, Odense University Hospital and Slagelse Hospital. The same method of analysis is applied to all four departments.

Nose and throat swabs are examined for the presence of MRSA: An enhancement broth [Tryptic soy broth supplemented with 2,5% NaCl, 3,5 mg/L cefoxitin and 20 mg/L aztreonam (Herlev Hospital, Herlev, Denmark)] is inoculated with 100 μL from both ESwab media and incubated at 35–37 °C for 16–24 h. Subsequently, a selective chromogenic medium [CHROMagar MRSA II agar (Becton Dickinson, Heidelberg, Germany)] is inoculated with 100 μL of the enhancement broth and incubated for an additional 42–48 h in atmospheric conditions. Possible isolates of *Staphylococcus aureus* are identified by mass spectrometry [(MALDI-TOF-MS: BioTyper/Bruker (Bruker Daltronics, Bremen, Germany) or Vitek MS (bioMérieux, Marcy-l’Etoile, France)] and screened for cefoxitin resistance according to the European Committee on Antimicrobial Susceptibility Testing (EUCAST, eucast.org) and for the presence of the *mecA*-gene by polymerase chain reaction (PCR). *mecA*-negative, cefoxitin resistant isolates are examined for the presence of the *mecC*-gene.

Rectum swabs are examined for the presence of VRE, ESBL, CPE and CD. VRE: 100 μL of the FecalSwab medium are transferred to an enhancement broth [Brain Heart Infusion Broth supplemented with vancomycin 4 mg/L and 60 mg/L aztreonam (Herlev Hospital)]. The enhancement broth is incubated at 35–37 °C for 16–24 h and 100 μL of the broth are subsequently transferred to a selective chromogenic medium [chromID VRE Agar (bioMérieux)] and incubated for further 42–48 h in atmospheric conditions. Possible isolates of *Enterococcus spp* are identified by MALDI-TOF-MS and all confirmed *Enterococcus spp* isolates are examined for the presence of the *vanA-* and *vanB*-gene by PCR. ESBL, CPE and CD: Three different selective chomogenic substrates [ESBL: CHROMagar ESBL bi-agar (Becton Dickinson), CPE: chromID CARBA SMART agar (bioMérieux) and CD: chromID C.difficile agar (bioMérieux)] are each inoculated with 100 μL of the FecalSwab medium and incubated at 35–37 °C under atmospheric conditions for 18–24 h (ESBL and CPE) or in an anaerobic atmosphere for 42–48 h (CD). Suspected isolates of *Enterobacteriaceae* (ESBL or CPE) or CD are identified by MALDI-TOF-MS. Possible ESBL isolates are phenotypically characterized according to EUCAST using the ROSCO Total ESBL Confirm Kit (ROSCO, Taastrup, Denmark). Possible CPE isolates are screened for meropenem susceptibility according to EUCAST and stored for later genotypic characterization. Possible CD isolates are examined for the presence of toxin A (*tcdA-*gene)*,* toxin B (*tcdB-*gene) and binary-toxin (*cdtA*-gene) by PCR*.* Binary-toxin positive CD isolates will be examined for genotypic markers for CD ribotype 027.

All obtained isolates of cefoxitin-resistant *Staphylococcus aureus*, *Enterococcus spp*, cefalosporin- and/or meropenem-resistant *Enterobacteriaceae* and *Clostridium difficile* will be stored at − 80 °C.

### Data collection, management, and analysis

#### Data entry

All patients will be provided with project running number, which will be used throughout the project in the interviews and processing of microbiological samples to secure anonymity. An ID file with the project running number and the personal registration number (PRN) number will allow tracing the patient information concerning use of antibiotics before the admission in the national medicine registries, and for returning test answers to those patients who requested this information.

The answers from the interviews are entered directly to an electronic questionnaire (SurveyXact®, Rambøll, Aarhus, Denmark) on tablet PCs used solely for the project. Data is transmitted directly to the central secured data file storage.

The microbiological samples are analyzed anonymously using project running numbers and microbiological data are recorded in the local laboratory information system [Aarhus, Odense and Slagelse: MADS (Aarhus University Hospital, Aarhus, Denmark [www.madsonline.dk]) and Aalborg: ADBact (Autonik Ab, Sweden)]. Data is extracted from these databases after the end of the study to the central database where the results are merged to the interview information. The results will not appear in the patients hospital file.

Microbiological test results are merged with the interview data using the project running number and data from the medicine registries are merged with the data set by using the PRN number.

After data cleansing and validation the final data file will be anonymized before data analysis.

Information concerning antibiotic treatment before the admission will be obtained from National Board of Health, medicine registries.

The data analyzes are conducted in STATA 14 in cooperation with a statistician.

The prevalence of the resistant bacteria are calculated at hospital level, regional level and national level and described with relation to residency (region and degree of urbanization), sex, age and risk factors.

In one part of the national aggregated data set, a screening model for identification of carrier stage of resistant bacteria is developed, based on the interview and medicine information. The model is then validated in another part of the dataset. The model building is based on the individual resistant bacteria, and includes univariate and multivariate logistic regression. Relevant indicators from well-defined domains are selected, tested for correlations and interactions as well as goodness of fit of the final model.

Non-participant analysis is performed. For missing data multiple imputation is used.

#### Monitoring

The daily inclusion of participants will be monitored by the steering committee and the numbers of inclusion will be instantly available for all the included centers on a home page.

No interim analysis will be made. The primary analysis of data will be performed by three members of the steering committee after the last patient has been included and all the microbiological analysis finalized. The results will be discussed and evaluated first in the steering committee and afterwards with all the included departments.

#### Protocol amendments

Important protocol modifications like changes in eligibility criteria or outcome will be communicated to the relevant parties, i.e. sponsor, trial registry and scientific ethical committee.

#### Confidentiality

The test material will be destroyed immediately after the sample has been analyzed. Bacterial isolates are stored for further characteristics.

All personal information and project samples are anonymized throughout the study process using a project ID running numbers and the results cannot be seen in the patient file or made available for the clinical care providers. The results of the final analysis are expected to be available only 4–6 months after the index hospitalization. A key file will be created that connects the project ID serial number with the patient’s civil registration number. It allows for further future analyzes and is necessary for any feedback to the included patients. The PRN key is registered in the registration form, which is saved on secured drive.

#### Access to data

Only the members of the steering committee will have access to the final trial dataset. Other researches may be granted access to the anonymized data for analysis on reasonable request to the corresponding author.

#### Ancillary and post-trial care

The data files will be handed to the Danish National Archieves upon completion of the study.

#### Dissemination policy

The results of the project will be presented in English in peer-reviewed articles in recognized scientific journals. A report is also prepared for use by the National Board of Health, including an overall assessment of the results and clinical implications, deviations between expected and actual results and achieved experience in the project.

The results of the project will also be disseminated through participation in academic and other conferences, as well as through the printed and electronic press.

The author panel for both scientific articles and the report to the Health Board will include the steering committee and one local researcher from each of the participating emergency departments and clinical microbiology departments in accordance with the Vancouver criteria. No professional writers will be used.

Positive, negative and inconclusive results will be published.

## Discussion

The study will provide the prevalence of colonized patients with resistant bacteria on arrival to the ED and variation in demographic patterns, and will develop a clinical tool to identify certain risk groups. This will enable the clinician to target antibiotic treatments and to reduce the in-hospital spreading of resistant bacteria. This knowledge is important for implementing and evaluating antimicrobial stewardships, screening and infection control strategies.

## Abbreviations

AB-RED: The antibiotic resistance in emergency departments in Denmark
CD: *Clostridium difficile*
CPE: Carbapenem resistant enterobacteria
ED: Emergency Department
ESBL: Extended-spectrum beta-lactamase
MRB: Multiresistant bacteria
MRSA: Methicillin resistant *Staphylococcus aureus*
PRN: Personal registration number
VRE: Vancomycin resistant enterococci
